# Effect of Buckwheat‐Containing Bread on Postprandial Glycemia, Appetite, Palatability, and Gastrointestinal Well‐Being

**DOI:** 10.1002/fsn3.4697

**Published:** 2025-05-26

**Authors:** Khoula Begum, Imran Khan, Mohammed H. Al‐Rizeiqi, Stuart K. Johnson, Ali Madi Almajwal

**Affiliations:** ^1^ Department of Human Nutrition The University of Agriculture Peshawar Peshawar Khyber Pakhtunkhwa Pakistan; ^2^ Department of Food Science and Nutrition, College of Agricultural and Marine Sciences Sultan Qaboos University Muscat Oman; ^3^ School of Public Health, Curtin Health Innovation Research Institute Faculty of Health Sciences, Curtin University Perth Western Australia; ^4^ Department of Community Health Sciences, College of Applied Medical Sciences King Saud University Riyadh Saudi Arabia

**Keywords:** appetite, buckwheat‐containing bread, control bread, glycemia

## Abstract

Pseudocereals like buckwheat are a significant source of health‐promoting bioactive components. Consumption of pseudocereals may positively modify biomarkers associated with chronic conditions. The main objective of the research was to determine how adding buckwheat‐containing bread to wheat flour affected gastrointestinal health, appetite, palatability, and postprandial glycemia in healthy individuals. In a randomized cross‐over trial, 20 healthy subjects were given either control bread (CB) or 50% buckwheat bread (BB) at breakfast after a 10‐ to 12‐h fast. The recommended daily bread intake was determined based on 50 g of available carbohydrates. Blood glucose levels and appetites were assessed before fasting and 15, 30, 45, 60, 90, and 120 min after bread intake. Standardized questionnaires were used to examine palatability and gastrointestinal well‐being. Buckwheat bread significantly lowered postprandial blood glucose levels compared to control bread. While peak glucose level occurred at 45 min for both, BB showed a smaller glucose increase. Repeated measure ANOVA confirmed a significant reduction at 30, 45, and 60 min. Overall, BB resulted in a lower incremental area under the curve (iAUC), indicating improved postprandial glycemic control. Additionally, BB demonstrated a trend toward enhancing satiety during the early postprandial phase (15–60 min) as reflected in Figure 2, potentially indicating a role in promoting satiety. The participants enjoyed all the bread‐containing buckwheat, and no gastrointestinal issues were noted. The study concluded that BB decreased appetite and enhanced postprandial glycemia compared to CB. Further research is suggested to investigate the process behind these reported impacts.

## Abstract 2

*Title*: Effects of Buckwheat bread on cardio‐metabolic biomarkers in Healthy Individuals. Study design: 20 healthy participants. *Method*: Randomized crossover trial. *Interventions*: Wheat bread as control bread (CB). *Treatment*: 50% buckwheat bread (BB). *Outcomes*: *Postprandial Glycemia*: Significant reduction in blood glucose levels at 30 min (*p* = 0.001) and 45 min (*p* = 0.003) after consuming BB as compared to CB. *Appetite*: Reduced hunger levels after BB consumption (*p* = 0.035 and *p* = 0.0347). *Palatability*: High enjoyment of buckwheat bread with no gastrointestinal issues reported. *Conclusions*: Buckwheat containing bread decrease appetite and enhances postprandial glycemia compared to conventional bread.

## 1 Introduction

Cardiometabolic health is a crucial aspect of overall well‐being, encompassing the interplay between cardiovascular health and metabolic function. Maintaining optimal cardiometabolic health is essential for reducing the risk of chronic diseases (Roth et al. 2020). Dietary modifications may be made to lower cardiometabolic diseases (Musaiger and Al‐Hazzaa 2012). According to (Marventano et al. 2017), several cardiometabolic diseases have been linked to increased intake of refined and processed foods and a fall in whole‐grain cereals, vegetables, and fruit consumption. Reduced postprandial blood glucose levels are associated with decreased calorie consumption, which may mitigate long‐term cardiometabolic risks (Augustin et al. 2015). Diabetes and cardiovascular illnesses have been associated with fasting and postprandial hyperglycemia because they cause oxidative stress, inflammation, and endothelial dysfunction (Blaak et al. 2012). According to Liu et al. (2018), consuming antioxidant‐rich foods may increase endogenous antioxidant levels.

According to several studies, eating whole grains has been associated with a lower risk of chronic diseases (Cho et al. 2013). Cereals, which contain grains, are the primary source of calories, minerals, and dietary fiber in the diet (McKevith 2004). Wheat is a cereal grain that is consumed the most widely on a global scale (Dhingra and Jood 2002). Although wheat flour has less bioactive compounds than other underutilized cereals, several research have explored the possibility of combining wheat flour with other grains and legumes that have more beneficial components, to improve the bioactive and nutritional profile of wheat flour. In a meal, cereals constitute the main source of calories, minerals, and dietary fiber (McKevith 2004). Underutilized cereals have demonstrated a remarkable capacity to partially replace other grains in meals, hence improving wheat flour's nutritional and bioactive component profile (El Sohaimy 2012). Eating underutilized cereals and grains has been responsible in several studies, lowering the chances of disease development (Cho et al. 2013).

Buckwheat is a polygonance family member produced in Gilgit‐Baltistan, Pakistan, as a minor crop. Buckwheat crops are suitable for single cropping sites in the far north of Pakistan because of their brief growing season. Buckwheat is a crop from cold, wet, temperate regions susceptible to high temperatures and hot, dry winds. Therefore, the geography and environment of Gilgit‐Baltistan favor its production (Facho et al. 2016). Buckwheat‐based grains are gluten free and have a number of health benefits, including the ability to lower cholesterol and possess anti‐inflammatory, anti‐cancer, and anti‐diabetic qualities (Noreen et al. 2021). Buckwheat's high nutritional content has drawn the attention of food processors and consumers in recent years (Giménez‐Bastida et al. 2018). Buckwheat groats possess a high starch content that makes them a viable substitute for traditional starchy crops including cassava, maize, and wheat (Bonafaccia, Marocchini, and Kreft 2003; Steadman et al. 2001).

In recent years, the rise in obesity and diabetes, there has been an increased interest in slowly digestible starch (SDS), or starch with a slow rate of digestion, among consumers and food industry (Lehmann and Robin 2007; Miao et al. 2015; Naser, Gruber, and Thomson 2006). Hence, SDS is slowly absorbed in the small intestine, lowers the postprandial blood glucose response, improves glycemic control and insulin sensitivity, delays the sensation of hunger, the lowers the risk of heart disease, stroke, and diabetes as well as the prevalence of obesity (Lehmann and Robin 2007; Miao et al. 2015).

The increasing demand for pseudocereals has been steadily expanding in the last several years. Their significance has increased as a substitute for conventional and commonly utilized basic ingredients for human nutrition (Haros and Schoenlechner 2017). Although pseudocereals not being members of Gramineae family, due to starchy endosperm, it may easily be ground into flour, which has a cereal‐like texture (Skrabanja et al. 2001). Buckwheat's high nutritional value makes it a useful ingredient in food compositions. Buckwheat is rich in protein, dietary fiber, antioxidants, phenolic compounds, vitamins, minerals, and resistance starch. The protective effects of buckwheat proteins, flavonoids, and thiamin‐binding proteins on blood pressure, cholesterol, and serum glucose levels have been shown in several researches (Bae et al. 2013; Vujić et al. 2014).

Conversely, buckwheat flour is rich in antioxidant polyphenols, which may help prevent oxidative stress. The health potential of buckwheat on biomarkers of cardiometabolic health is well established. To make functional bread that is high in nutrients and to investigate how it affects gastrointestinal symptoms, hunger, and blood glucose levels in healthy adults, buckwheat flour has been combined with white bread in this study.

## 2 Methodology

The study was conducted in the laboratory of the University of Agriculture Peshawar, Khyber Pakhtunkhwa.

### 2.1 Selection of Individuals

Twenty healthy individuals (10 male and 10 female) aged between 18 and 30 years with average weight and height were selected for the study through personal communication. Informed written consent was acquired from each person. The current research included healthy individual's ages 18–30 years. They also had a normal BMI (18.5–25.0 kg/m^2^) and a fasting blood glucose level less than 110 mg/dL. Individuals in the research were excluded if they had a history of type 1 or type 2 diabetes, hyperinsulinemia, gastrointestinal disease, eating disorders, smokers, pregnant, nursing women, and breakfast skippers. Individuals with any medicine that influences insulin level, hunger, or weight were excluded from the study. Before participating in the study, individuals were assessed using the health screening questionnaire. Individuals were given thorough explanations of the research procedures and were allowed to learn about any investigations they had. Additionally, individuals had the freedom to leave research whenever they wanted. The Helsinki Declaration standards were followed when conducting the study. The Department of Human Nutrition, Human Research Ethics Committee (HN‐HRC/2018‐2021) of the University of Agriculture, Peshawar, approved the study protocol.

The sample size of the research was determined on the previous study basis (Khan et al. 2014). The research showed that 20 individuals were required to find a 24% change in total plasma polyphenol levels with a power of 80% and an α value of ≤ 0.05.

### 2.2 Research Design

A total of 32 participants were screened, of which 24 qualified eligibility criteria of the study. The eligible individuals entered the randomization part of the research, and 20 completed the study. A randomized controlled crossover design was used for the research. Every individual needed to attend two sessions. The washout time (test samples) should be separated by 1–2 weeks. The participants received one or two test breads (control or treatment) during each session at breakfast. The distribution of the test bread included randomly chosen digital numbers. The individuals in the study follow a regular diet and refrain from engaging in physically demanding activities during the study period. The individuals were instructed to eat the same meal before the research day to reduce variance. The individual were told to complete their dinner by 10.00 pm, and then they continued the fast. Water was permitted for them to consume until the following morning. Primary assessments were recorded, such as height, weight, and waist circumference. Before the fasting blood sample was taken, the individuals were told to lie supine and relax. A Labeled Magnitude (LMS) was used to record the subjects' satiety sensations while they were fasting, and capillary blood samples were obtained using a lancet device and the finger prick technique. Therefore, individuals were given test bread. Within 10–15 min, individuals finished their test bread. Then, blood glucose was tested at times 15, 30, 45, 60, 90, and 120 min after intake of test bread by using the finger prick method. Individuals recorded their LMS satiety sensation after each blood collection. Individuals waited 2 h to eat or drink.

### 2.3 Baseline Assessment

The weight of each individual was ascertained using a digital scale. Taking off shoes and dressing in a minimum number of clothes were the criteria for calculating weight. The stadiometer was used to measure each height closest to 0.1 cm. With a tape (nonstretchable) and standard technique, the waist circumference was recorded closest to 0.1 cm. A manual mercurial sphygmomanometer (Yamasu; 600, Kenzmedico. Co. LTD, Tokyo, Japan) was used to assess blood pressure.

### 2.4 Blood Glucose Analysis

Blood samples were obtained using a lancet device using the finger prick method to assess blood glucose. Protocols for testing were developed using standard glucose techniques (Brouns et al. 2005). To increase blood circulation, the individuals warmed their hands; the tips to the base of the fingers were massaged. To avoid dilution of plasma, fingers were not squeezed. Before pricking, the finger was cleaned using an alcoholic swab. Blood samples were collected at 0 min (fasting) and then at 15, 30, 45, 60, 90, and 120 min using the Accu Check blood glucose analyzer. Throughout the study, the glucometer was not changed to minimize “intra‐subject variation” the same glucometer was used. The readings were recorded using a blood glucose recording sheet.

### 2.5 Satiety Assessments

A magnitude satiety scale (LMS) (19 cm) was used (Mohd‐Kasim et al. 2008). LMS with ZERO at the center point is identified with the label “Neither Hungry nor Full,” which has a negative score of −9.5 cm on the left side, “Greatest Imaginable Hunger,” while on the right side, “Greatest Imaginable Fullness,” a positive score of 9.5 cm of the scale. Subjects indicated their level of hunger or fullness by making a mark on the scale. The vertical line distance from the center is measured, with a closest value of 0.1 cm. Satiety assessments were planned at fasting (0 min) and then at 15, 30, 45, 60, 90, 120 min.

### 2.6 Palatability Assessments

Individual palatability was assessed on a nine‐point hedonic scale in terms of the bread's look, flavor, texture, and acceptability. The subjects were asked to mark their perception of the bread anywhere along the scale.

### 2.7 Gastrointestinal Symptoms

Digestive complaints, such as nausea, heartburn, vomiting, and stomach discomfort, were noted at 0, 30, 60, 90, and 120 min after taking the bread samples.

### 2.8 Statistical Analysis

SPSS was used to analyze the research data. Blood glucose, GI, and satiety responses were examined using a two‐way repeated ANOVA with post hoc Bonferroni analysis. A paired *t*‐test was used to determine changes based on the interaction effect at specific time points. A statistically significant value was *p* < 0.05.

## 3 Results

Twenty healthy subjects 10 male and 10 female were selected for the current research. The baseline characteristics of the individuals are presented in Table 1, which shows that the average age of individuals was 24 years. According to research, their body mass index falls under the normal category, whereas waist circumference, fasting glucose, and blood pressure shows a healthy individuals selection.

**TABLE 1:** Baseline characteristics of study subjects (*n* = 20).^a^

Variable	Healthy
M/F	10/10
Age (years)	24.35 ± 2.39
Weight (kg)	63.35 ± 8.28
Height (m)	165.45 ± 7.79
BMI (kg/m^2^)	23.01 ± 2.31
Waist circumference (cm)	79.77 ± 6.99
Fasting plasma glucose (mg/dL)	90.50 ± 5.21
Systolic blood pressure (mm Hg)	109.65 ± 12.17
Diastolic blood pressure (mm Hg)	77.25 ± 9.44

*Note:* The mean value of three measurements performed during the OGTT.

Abbreviation: BMI, body mass index.

^a^ Values are means ± SEM.

### 3.1 Blood Glucose

**FIGURE 1:**
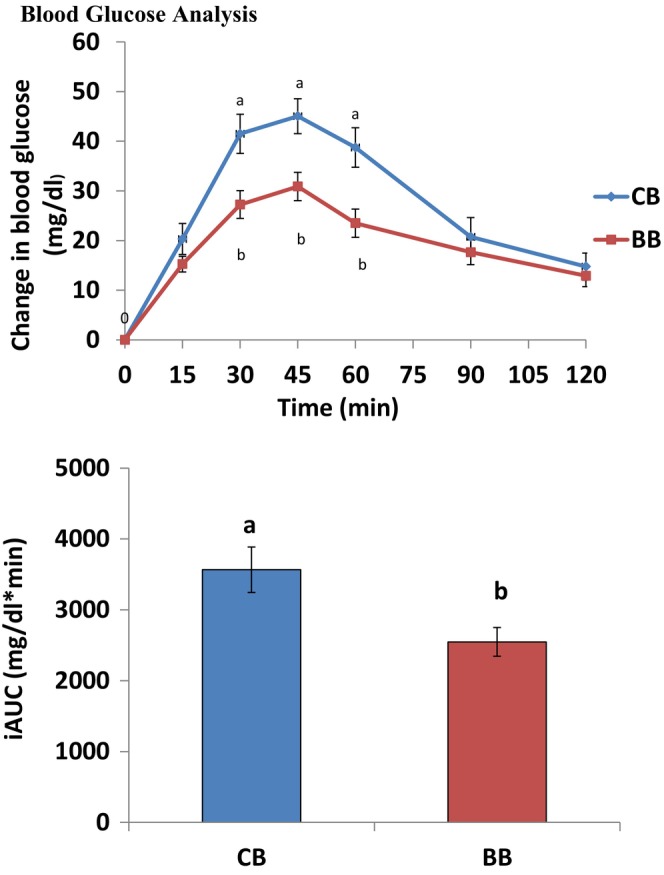
Mean (± SEM) changes from baseline in blood glucose and incremental areas under the curves (AUC) in healthy subjects (*n* = 20) after consumption of test bread. BB, buckwheat bread; CB, control bread. Different letters shows that blood glucose level reduce but not significantly.

Figure 1 displays the postprandial glucose level with the respective incremental area under the curve. Using repeated–measures ANOVA, a post hoc pairwise comparison reveals that bread containing 50% BB significantly lowered glucose levels (*p* = 0.001) compared to control bread.

### 3.2 Satiety Response

**FIGURE 2:**
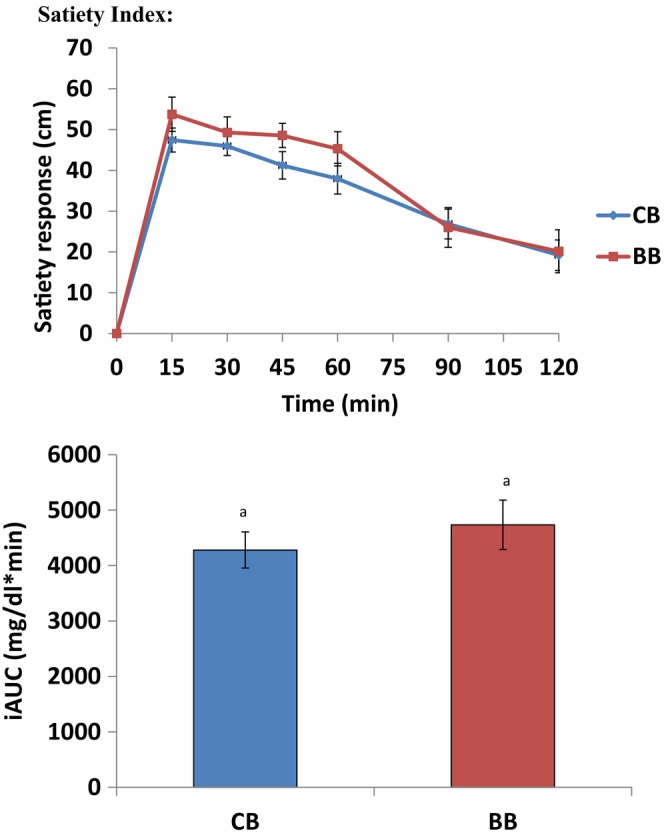
Mean (± SEM) changes from baseline in satiety response and incremental areas under the curves (iAUCs) in healthy subjects (*n* = 20) after consumption of test meals. BB, buckwheat bread; CB, control bread. Different letters shows that blood glucose level reduce but not significantly.

Figure 2 displays the postprandial satiety responses expressed as appetite responses and the corresponding incremental area under the curve.

### 3.3 Incremental Peak Glucose of Bread Samples

When buckwheat flour was added, the bread glycemic index dropped dramatically (*p* = 0.019), going from 100 to 80.84 on the bread scale and from 71 to 57.1 on the glucose scale (Table 2).

**TABLE 2:** Incremental peak glucose of test bread.*

Test meal	Peak time	Incremental peak glucose**	Glycemic profile**	Glycemic index (bread scale)**	Glycemic index (glucose scale)**	Satiety index
CB	43.50 ± 3.59	51.7 ± 3.33^a^	2.38 ± 0.38^b^	100.00 ± 0.00^a^	71.00 ± 0.00^a^	100.00 ± 0.00
BB	49.50 ± 3.93	36.25 ± 2.82^b^	3.15 ± 0.19^a^	80.35 ± 8.34^b^	57.05 ± 5.92^b^	118.59 ± 10.03

Abbreviations: BB, buckwheat bread; CB, control bread.

* All values are means ± SEMs (*n* = 20).

** Values in the same column with different superscript letters are significantly different, *p* < 0.05 (paired *t*‐test).

### 3.4 Palatability Measurements

A nine‐point hedonic scale was used to evaluate the bread's palatability regarding look, flavor, texture, and overall acceptability. Control bread's palatability parameters ranged from 7.45 to 7.55, whereas buckwheat bread was found to have a range of 6.8–7.02. The results showed that with the exception of appearance, there was no statistically significant difference between the two breads. The individuals found both breads tasty and acceptable, with no significant difference in palatability (Table 3).

**TABLE 3:** Acceptability parameters of the test bread.*

Test meal	Appearance	Texture	Flavor	Overall acceptance
CB	7.45 ± 0.28^a^	7.30 ± 0.30	7.35 ± 0.24	7.55 ± 0.24
BB	6.85 ± 0.17^b^	6.95 ± 0.23	7.05 ± 0.23	7.20 ± 0.37

*Note:* The different superscripts shows that the control bread and buckwheat bread were statistically not significant.

Abbreviations: BB, buckwheat bread; CB, control bread.

* Values are means ± SEM. Values for acceptability parameters are not significantly different. *p* < 0.05 (paired *t*‐test).

### 3.5 Gastrointestinal Symptoms

**FIGURE 3:**
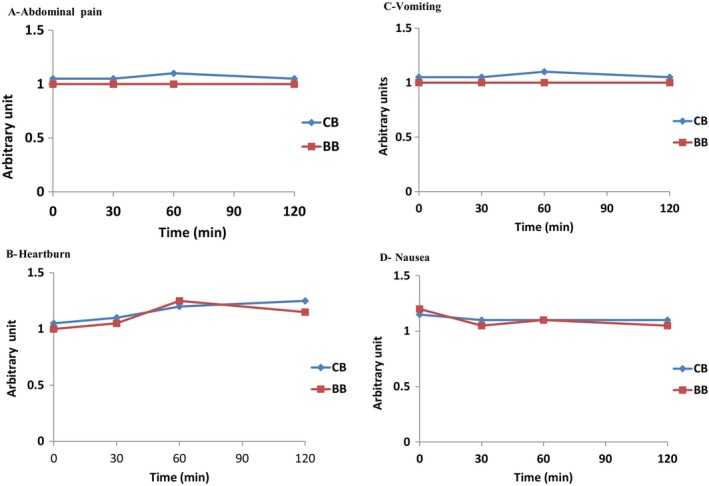
Mean (± SEM) responses of healthy subjects (*n* = 20). Two‐way repeated measure ANOVA, followed by paired *t*‐test, Bonferroni adjustment (*p* < 0.05). (A) abdominal pain; (B) heartburn; (C) vomiting; (D) nausea.

Individuals were investigated for gastrointestinal symptoms, including abdominal pain, vomiting, heartburn, and nausea. The signs mentioned above were noted at time 0 (shortly before consuming) and 30, 60, 90, and 120 min after consuming bread. The individuals to calculate these symptoms' strength, occurrence, and rating as “none, mild, moderate, quite a lot, severe, very severe, and unbearable.” All individuals consumed the bread, and no adverse signs and symptoms were observed in gastrointestinal symptoms, including abdominal pain, vomiting, heartburn, and nausea (Figure 3).

## 4 Discussion

The cross‐over randomized trial design aimed to investigate the impact of buckwheat‐incorporated wheat bread on specific biochemical markers in 20 healthy individuals. It was hypothesized that buckwheat‐containing bread would improve blood glucose levels, satiety index, bread palatability, and related gastrointestinal problems. Dietary ways must overcome the issue of creating valuable consumer food and include additional nutraceuticals and functional ingredients without sacrificing flavor or other sensory aspects (Tuorila and Cardello 2002).

The incorporation of buckwheat into bread has been shown to influence glycemic control, insulin response and satiety in healthy individuals. Different studies indicate that consuming bread made with buckwheat has been shown to change the postprandial responses of gastrointestinal hormones, which are essential for controlling food intake and glucose metabolism. For instance, a study comparing the effect of whole‐grain buckwheat flour vs. rice flour on healthy participants found that buckwheat consumption significantly changed the levels of satiety hormones glucagon‐like‐peptide‐1 (GLP‐1) and pancreatic polypeptide, but there were no significant changes in postprandial glucose or insulin levels (Stringer et al. 2013; Begum et al. 2024; Yin et al. 2022).

The incorporation of soluble dietary fiber and polyphenols to bread made with buckwheat has a major impact on insulin responses, glycemic management, and satiety in healthy individuals. Buckwheat and other soluble dietary fibers decrease the absorption of carbohydrates and improve insulin sensitivity, which can help regulate postprandial blood glucose levels. Research shows that soluble fiber‐rich food had lower glycemic indices (GI) than high‐GI foods, which is associated with less insulin spikes and longer periods of satiety (Holt et al. 1992). Most foods containing resistant starch have a low glycemic index because low glycemic index foods control blood sugar, decrease obesity, and lower the risk of heart disease, buckwheat may be used to treat such chronic disease (Skrabanja et al. 2001; Zhang et al. 2012; Wronkowska, Soral‐Śmietana, and Krupa‐Kozak 2010). Studies on 10 healthy participants consumed white bread, 50% supplemented with buckwheat flour, and cooked buckwheat groats. The study's findings showed that people who consumed buckwheat products, especially buckwheat groats produced less insulin and had lower postprandial plasma glucose levels than those who consumed white wheat bread (Su‐Que et al. 2013). Randomly selected 10 diabetics, who were shown to have a 51% lower plasma glucose level after consuming buckwheat bread. Due to antinutritional elements like polyphenols and enzyme inhibitors, the body may be more difficult to digest buckwheat compared to wheat and legumes. The digestion delays help in regulating blood glucose levels. With healthy diet, buckwheat consumption positively affects insulin levels (Qiu et al. 2016).

In a research conducted on healthy adults who have type 2 diabetes, individuals were given foods made from white flour and buckwheat flour for a week. Consequently, no changes were observed in the concentration of insulin and glucose. However, a modulation of gastrointestinal satiety hormones was documented after consumption of a diet made from buckwheat flour in both healthy and diabetic individuals (Stringer et al. 2013). According to (Kreft et al. 2022), eating buckwheat kasha enhances insulin response and prolongs the feeling of fullness. Food made with buckwheat contains metabolites such as tannins and quercetin that prevent the digestion of carbohydrates. Finally, compared to other plant and animal protein‐rich meals, buckwheat was found to be particularly effective at reducing hunger and promoting satiety (Neacsu et al. 2022).

Furthermore, buckwheat's polyphenols may intensify these benefits by enhancing endothelial function and lowering oxidative stress, both of which are advantages for metabolic health (Bolton, Heaton, and Burroughs 1981).

The consumption of buckwheat‐containing has been shown to influence satiety hormones like ghrelin and peptide YY (PYY). Research shows that eating a lot of fiber can lead to increased PYY levels, which promote satiety. In a controlled trial, participants who consume buckwheat bread expressed feeling more full than those consuming traditional wheat bread, indicating that the fiber level is important in controlling appetite (Kempf et al. 2023). Compared to regular bread, consuming buckwheat bread decreased postprandial insulin levels in studies including healthy participants. This effect is ascribed to the combined action of polyphenols and dietary fibers, which inhibit the digestion and absorption of carbohydrates (Yin et al. 2022).

The bioaccessibilty of these phenolics during digestion affects buckwheat breads antioxidant potential. Studies show that after gastrointestinal digestion, up to 90% of total phenolics can become bioavailable, greatly increasing their antioxidant capacity. This ability has been measured using the global antioxidant response (GAR) approach, which shows that buckwheat‐enriched breads have significantly higher GAR values than regular wheat bread (Szawara‐Nowak, Bączek, and Zieliński 2016).

Polyphenols are known to affect gastrointestinal health through different mechanisms, including modulate gut microbiota, reducing inflammation, and improving mucosal barrier function. By suppressing harmful strains of gut bacteria and encouraging the growth of healthy ones, polyphenols in the diet can enhance gut health in general (Wan, Co, and El‐Nezami 2021). In studies involving buckwheat‐containing bread, participants reported improvements in digestive health markers. Buckwheat's soluble fiber level is also essential for encouraging regular bowel motions and avoiding constipation (Verardo et al. 2018).

The impact of polyphenolic compounds on insulin sensitivity and blood glucose levels has been a significant attention. Buckwheat has been demonstrated to have positive benefits on glycemic control due to its low glycemic index (GI) and high fiber content. Research indicate that buckwheat‐based meals have been shown to reduce postprandial blood glucose levels compared to refines grain‐based meals. Buckwheat polyphenols may improve endothelial function and lower inflammation, which may increase insulin sensitivity. In a controlled study, participants consuming buckwheat‐enriched bread had lower insulin levels than those who were on traditional wheat bread. This suggests that incorporating buckwheat in the diet might be an effective strategy for managing blood glucose levels, particularly for individuals at risk for type II diabetes (Lin et al. 2009).

Buckwheat can treat chronic diseases, such as low glycemic index foods, control blood sugar, and decrease obesity (Skrabanja et al. 2001). According to (Cummings and Stephen 2007), they are subjectively assessed for tolerance, and the interpretation of the gastrointestinal parameters may differ among the participants. In the present study, it was expected that BWB may experience some gastrointestinal symptoms related to the increased fiber content of the test bread. Although gastrointestinal symptoms may not harm an individual's health, they can hurt well‐being and decrease consumer acceptability (Jl and Green 2007). However, in this trial, no gastrointestinal problems were statistically significant.

Overall, our results underline buckwheat importance as a functional food in controlling metabolic health and the potential advantages of including it in dietary patterns to improve glycemic control and promote satiety in healthy individuals.

### 4.1 Limitations

The research concluded that consuming buckwheat‐containing bread may modify glucose responses. Future research is now suggested to explore the mechanisms behind the hypoglycemia and satiety‐enhancing effects. The current research concentrates on short‐term impacts; further research is needed to determine the long‐term implications. Registry of clinical trial is one of limitation of our study.

## 5 Conclusion

The conclusion suggests that replacing wheat with underutilized cereals like buckwheat may lower the risk of cardiometabolic diseases due to its high dietary fiber and polyphenolic content. In healthy individuals, the intake of buckwheat‐containing bread is expected to improve glycemic and satiety responses without causing gastrointestinal symptoms.

## Author Contributions

Research materials, preparing written reviews and edits, formal analysis, self‐funding, methods, research, validation of supervision, and examination are formal. The current study work has never been submitted to other journals except for a PhD thesis.

## Conflicts of Interest

The authors declare no conflicts of interest.

## Supporting information


Appendix

## Data Availability

If needed, research materials and data are available.

## References

[fsn34697-bib-0001] Augustin, L. S. , C. W. Kendall , D. J. Jenkins , et al. 2015. “Glycemic Index, Glycemic Load and Glycemic Response: An International Scientific Consensus Summit From the International Carbohydrate Quality Consortium (ICQC).” Nutrition, Metabolism, and Cardiovascular Diseases 25, no. 9: 795–815. 10.1016/j.numecd.2015.05.005 26160327

[fsn34697-bib-0002] Bae, I. Y. , H. I. Lee , A. Ko , and H. G. Lee . 2013. “Substituting Whole Grain Flour for Wheat Flour: Impact on Cake Quality and Glycemic Index.” Food Science and Biotechnology 22: 1–7.

[fsn34697-bib-0003] Begum, K. , I. Khan , A. Wali , et al. 2024. “Buckwheat Containing‐Bread: A Scientific Inquiry Into Insulin, Polyphenols, Antioxidants Status, and Oxidative Stress Markers in Type‐II Diabetic Individuals.” Frontiers in Sustainable Food Systems 8: 1440053.

[fsn34697-bib-0004] Blaak, E. , J. M. Antoine , D. Benton , et al. 2012. “Impact of Postprandial Glycaemia on Health and Prevention of Disease.” Obesity Reviews 13, no. 10: 923–984. 22780564 10.1111/j.1467-789X.2012.01011.x PMC3494382

[fsn34697-bib-0005] Bolton, R. P. , K. W. Heaton , and L. F. Burroughs . 1981. “The Role of Dietary Fiber in Satiety, Glucose, and Insulin: Studies With Fruit and Fruit Juice.” American Journal of Clinical Nutrition 34, no. 2: 211–217. 6259919 10.1093/ajcn/34.2.211

[fsn34697-bib-0006] Bonafaccia, G. , M. Marocchini , and I. Kreft . 2003. “Composition and Technological Properties of the Flour and Bran From Common and Tartary Buckwheat.” Food Chemistry 80, no. 1: 9–15.

[fsn34697-bib-0007] Brouns, F. , I. Bjorck , K. N. Frayn , et al. 2005. “Glycaemic Index Methodology.” Nutrition Research Reviews 18, no. 1: 145–171. 19079901 10.1079/NRR2005100

[fsn34697-bib-0008] Cho, S. S. , L. Qi , G. C. Fahey , and D. M. Klurfeld . 2013. “Consumption of Cereal Fiber, Mixtures of Whole Grains and Bran, and Whole Grains and Risk Reduction in Type 2 Diabetes, Obesity, and Cardiovascular Disease.” American Journal of Clinical Nutrition 98, no. 2: 594–619. 23803885 10.3945/ajcn.113.067629

[fsn34697-bib-0009] Cummings, J. H. , and A. M. Stephen . 2007. “Carbohydrate Terminology and Classification.” European Journal of Clinical Nutrition 61, no. Suppl 1: S5–S18. 17992187 10.1038/sj.ejcn.1602936

[fsn34697-bib-0010] Dhingra, S. , and S. Jood . 2002. “Organoleptic and Nutritional Evaluation of Wheat Breads Supplemented With Soybean and Barley Flour.” Food Chemistry 77, no. 4: 479–488.

[fsn34697-bib-0011] El Sohaimy, S. 2012. “Functional Foods and Nutraceuticals‐Modern Approach to Food Science.” World Applied Sciences Journal 20, no. 5: 691–708.

[fsn34697-bib-0012] Facho, Z. H. , Farhatullah , I. Khalil , N. Ullah Khan , and S. Ali . 2016. “Morphological Characterization and Estimation of Genotype × Environment Interaction of Indigenous Buckwheat Germplasm Collected From Gilgit Baltistan Pakistan.” Pakistan Journal of Botany 48: 2391–2398.

[fsn34697-bib-0013] Giménez‐Bastida, J. , J. M. Laparra‐Llopis , N. Baczek , and H. Zielinski . 2018. “Buckwheat and Buckwheat Enriched Products Exert an Anti‐Inflammatory Effect on the Myofibroblasts of Colon CCD‐18Co.” Food & Function 9, no. 6: 3387–3397. 29870039 10.1039/c8fo00193f PMC6597957

[fsn34697-bib-0014] Haros, C. M. , and R. Schoenlechner . 2017. Pseudocereals: Chemistry and Technology. New York, NY: John Wiley & Sons.

[fsn34697-bib-0015] Holt, S. , J. Brand , C. Soveny , and J. Hansky . 1992. “Relationship of Satiety to Postprandial Glycaemic, Insulin and Cholecystokinin Responses.” Appetite 18, no. 2: 129–141. 1610161 10.1016/0195-6663(92)90190-h

[fsn34697-bib-0016] Jl, S. , and H. Green . 2007. “Dietary Fibre and Satiety.” Nutrition Bulletin 32: 32–42.

[fsn34697-bib-0017] Kempf, K. , M. Röhling , H. Kolb , and S. Martin . 2023. “Impact of a Low‐Insulin‐Stimulating Bread on Weight Development—A Real Life Randomised Controlled Trial.” Nutrients 15, no. 5. 10.3390/nu15051301 PMC10004839 36904300

[fsn34697-bib-1001] Khan, I. , A. M. Yousif , S. K. Johson , and S. Gamlath . 2014. “Effect of Sorgham Flour Addition on *In Vitro* Starch Digestibilty, Cooking Quality, and Consumer Acceptibilty of Durum Wheat Pasta.” Journal of Food Science 79, no. 8: S1560–S1567. 25047068 10.1111/1750-3841.12542

[fsn34697-bib-0018] Kreft, I. , M. Germ , A. Golob , et al. 2022. “Phytochemistry, Bioactivities of Metabolites, and Traditional Uses of Fagopyrum tataricum .” Molecules 27, no. 20: 7101. 36296694 10.3390/molecules27207101 PMC9611693

[fsn34697-bib-0019] Lehmann, U. , and F. Robin . 2007. “Slowly Digestible Starch–Its Structure and Health Implications: A Review.” Trends in Food Science & Technology 18, no. 7: 346–355.

[fsn34697-bib-0020] Lin, L.‐Y. , H. M. Liu , Y. W. Yu , S. D. Lin , and J. L. Mau . 2009. “Quality and Antioxidant Property of Buckwheat Enhanced Wheat Bread.” Food Chemistry 112, no. 4: 987–991.

[fsn34697-bib-0021] Liu, Z. , Z. Ren , J. Zhang , et al. 2018. “Role of ROS and Nutritional Antioxidants in Human Diseases.” Frontiers in Physiology 9: 477. 29867535 10.3389/fphys.2018.00477 PMC5966868

[fsn34697-bib-0022] Marventano, S. , C. Vetrani , M. Vitale , J. Godos , G. Riccardi , and G. Grosso . 2017. “Whole Grain Intake and Glycaemic Control in Healthy Subjects: A Systematic Review and Meta‐Analysis of Randomized Controlled Trials.” Nutrients 9, no. 7: 769. 28753929 10.3390/nu9070769 PMC5537883

[fsn34697-bib-0023] McKevith, B. 2004. “Nutritional Aspects of Cereals.” Nutrition Bulletin 29, no. 2: 111–142.

[fsn34697-bib-0024] Miao, M. , B. Jiang , S. W. Cui , T. Zhang , and Z. Jin . 2015. “Slowly Digestible Starch—A Review.” Critical Reviews in Food Science and Nutrition 55, no. 12: 1642–1657. 24915311 10.1080/10408398.2012.704434

[fsn34697-bib-0025] Mohd‐Kasim, Z. , D. R. Greenway , N. A. Caffin , B. R. D'Arcy , and M. Gidley . 2008. “Application of Labeled Magnitude Satiety Scale in a Linguistically‐Diverse Population.” Food Quality and Preference 19: 574–578.

[fsn34697-bib-0026] Musaiger, A. O. , and H. M. Al‐Hazzaa . 2012. “Prevalence and Risk Factors Associated With Nutrition‐Related Noncommunicable Diseases in the Eastern Mediterranean Region.” International Journal of General Medicine 5: 199–217. 22399864 10.2147/IJGM.S29663 PMC3295618

[fsn34697-bib-0027] Naser, K. A. , A. Gruber , and G. Thomson . 2006. “The Emerging Pandemic of Obesity and Diabetes: Are We Doing Enough to Prevent a Disaster?” International Journal of Clinical Practice 60, no. 9: 1093–1097. 16939551 10.1111/j.1742-1241.2006.01003.x

[fsn34697-bib-0028] Neacsu, M. , S. de Lima Sampaio , H. E. Hayes , et al. 2022. “Nutritional Content, Phytochemical Profiling, and Physical Properties of Buckwheat (Fagopyrum esculentum) Seeds for Promotion of Dietary and Food Ingredient Biodiversity.” Crops 2, no. 3: 287–305.

[fsn34697-bib-0029] Noreen, S. , B. Rizwan , M. Khan , and S. Farooq . 2021. “Health Benefits of Buckwheat (Fagopyrum esculentum), Potential Remedy for Diseases, Rare to Cancer: A Mini Review.” Infectious Disorders Drug Targets 21, no. 6: e170721189478. 33357186 10.2174/1871526520999201224122605

[fsn34697-bib-0030] Qiu, J. , Y. Liu , Y. Yue , Y. Qin , and Z. Li . 2016. “Dietary Tartary Buckwheat Intake Attenuates Insulin Resistance and Improves Lipid Profiles in Patients With Type 2 Diabetes: A Randomized Controlled Trial.” Nutrition Research 36, no. 12: 1392–1401. 27919453 10.1016/j.nutres.2016.11.007

[fsn34697-bib-0031] Roth, G. A. , G. A. Mensah , C. O. Johnson , et al. 2020. “Global Burden of Cardiovascular Diseases and Risk Factors, 1990–2019: Update From the GBD 2019 Study.” Journal of the American College of Cardiology 76, no. 25: 2982–3021. 33309175 10.1016/j.jacc.2020.11.010 PMC7755038

[fsn34697-bib-0032] Skrabanja, V. , H. G. M. Liljeberg Elmståhl , I. Kreft , and I. M. E. Björck . 2001. “Nutritional Properties of Starch in Buckwheat Products: Studies In Vitro and In Vivo.” Journal of Agricultural and Food Chemistry 49, no. 1: 490–496. 11170616 10.1021/jf000779w

[fsn34697-bib-0033] Steadman, K. , M. S. Burgoon , B. A. Lewis , S. E. Edwardson , and R. L. Obendorf . 2001. “Buckwheat Seed Milling Fractions: Description, Macronutrient Composition and Dietary Fibre.” Journal of Cereal Science 33, no. 3: 271–278.

[fsn34697-bib-0034] Stringer, D. M. , C. G. Taylor , P. Appah , H. Blewett , and P. Zahradka . 2013. “Consumption of Buckwheat Modulates the Post‐Prandial Response of Selected Gastrointestinal Satiety Hormones in Individuals With Type 2 Diabetes Mellitus.” Metabolism 62, no. 7: 1021–1031. 23485142 10.1016/j.metabol.2013.01.021

[fsn34697-bib-0035] Su‐Que, L. , M. Ya‐Ning , L. Xing‐Pu , Z. Ye‐Lun , S. Guang‐Yao , and M. Hui‐Juan . 2013. “Effect of Consumption of Micronutrient Enriched Wheat Steamed Bread on Postprandial Plasma Glucose in Healthy and Type 2 Diabetic Subjects.” Nutrition Journal 12: 1–7. 23680007 10.1186/1475-2891-12-64 PMC3679746

[fsn34697-bib-0036] Szawara‐Nowak, D. , N. Bączek , and H. Zieliński . 2016. “Antioxidant Capacity and Bioaccessibility of Buckwheat‐Enhanced Wheat Bread Phenolics.” Journal of Food Science and Technology 53, no. 1: 621–630. 26787981 10.1007/s13197-015-2074-y PMC4711486

[fsn34697-bib-0037] Tuorila, H. , and A. Cardello . 2002. “Consumer Responses to an Off‐Flavor in Juice in the Presence of Specific Health Claims.” Food Quality and Preference 13: 561–569.

[fsn34697-bib-0038] Verardo, V. , V. Glicerina , E. Cocci , A. G. Frenich , S. Romani , and M. F. Caboni . 2018. “Determination of Free and Bound Phenolic Compounds and Their Antioxidant Activity in Buckwheat Bread Loaf, Crust and Crumb.” LWT 87: 217–224.

[fsn34697-bib-0039] Vujić, L. , D. V. Čepo , B. Šebečić , and I. V. Dragojević . 2014. “Effects of Pseudocereals, Legumes and Inulin Addition on Selected Nutritional Properties and Glycemic Index of Whole Grain Wheat‐Based Biscuits.” Journal of Food & Nutrition Research 53, no. 2.

[fsn34697-bib-0040] Wan, M. L. Y. , V. A. Co , and H. El‐Nezami . 2021. “Dietary Polyphenol Impact on Gut Health and Microbiota.” Critical Reviews in Food Science and Nutrition 61, no. 4: 690–711. 32208932 10.1080/10408398.2020.1744512

[fsn34697-bib-0041] Wronkowska, M. , M. Soral‐Śmietana , and U. Krupa‐Kozak . 2010. “Buckwheat, as a Food Component of a High Nutritional Value, Used in the Prophylaxis of Gastrointestinal Diseases.” Buckwheat 2: 64–70.

[fsn34697-bib-0042] Yin, X. , S. Liu , X. Zhang , et al. 2022. “Hypoglycemic Effects and Mechanisms of Buckwheat‐Oat‐Pea Composite Flour in Diabetic Rats.” Food 11, no. 23. 10.3390/foods11233938 PMC9739861 36496746

[fsn34697-bib-0043] Zhang, Z.‐L. , M. L. Zhou , Y. Tang , et al. 2012. “Bioactive Compounds in Functional Buckwheat Food.” Food Research International 49, no. 1: 389–395.

