# Supplementary material for: Effect of Buckwheat‐Containing Bread on Postprandial Glycemia, Appetite, Palatability, and Gastrointestinal Well‐Being
Source: Food Sci Nutr. 2025 May 26;13(6):e4697. doi: 10.1002/fsn3.4697 (PMC12106358; doi:10.1002/fsn3.4697)
Supplement: Supplementary file 1 — Appendix [file FSN3-13-e4697-s001.docx]

**Appendix-I**

**PALATABILITY QUESTIONNARE**

Subject #:____________Sample Code: ___________ Date:_____________

Please rate the food in terms of the following characteristics- study II

| Characteristic | Dislike extremely | Dislike very much | Dislike moderately | Dislike slightly | Neither like nor dislike | Like slightly | Like moderately | Like very much | Like extremely |
| --- | --- | --- | --- | --- | --- | --- | --- | --- | --- |
| Appearance |  |  |  |  |  |  |  |  |  |
| Texture |  |  |  |  |  |  |  |  |  |
| Flavor |  |  |  |  |  |  |  |  |  |
| Overall acceptance |  |  |  |  |  |  |  |  |  |

**Appendix-II**

**GASTROINTESTINAL SYMPTOMS QUESTIONNARE**

**Participant #:______________Date:_______________**

**Sample Code:____________ Time:__________________**

Do you experience any of the following symptoms? If yes, describe the intensity of the symptoms?

| Symptoms | Absent | Mild | Moderate | Quite a lot | Severe | Very Severe | Unbearable |
| --- | --- | --- | --- | --- | --- | --- | --- |
| Abdominal pain |  |  |  |  |  |  |  |
| Heartburn |  |  |  |  |  |  |  |
| Vomiting |  |  |  |  |  |  |  |
| Nausea |  |  |  |  |  |  |  |

**Nutrient composition of breads**

Two different kinds of bread were utilized in this investigation. Refined wheat flour was used to make the control bread (CB). The other was treatment bread, prepared with (50 %) buckwheat and refined flour. The bread was followed by the straight dough method [24]. The dietary fiber and phenolic concentration was more significant in buckwheat bread than in control bread; the nutritional profile of both breads was equal.

**Table 1: Nutrient composition of test breads**

| Ingredients | CB | BB |
| --- | --- | --- |
| Bread (g) | 103 | 105 |
| Water (g) | 247 | 245 |
| Total weight (g) | 350 | 350 |
| Energy (Kcal) | 288.66 | 304.09 |
| Av. CHO (g) | 50 | 50 |
| Protein (g) | 10.60 | 9.16 |
| Fats (g) | 5.14 | 6.05 |
| Dietary fiber (g)  Total phenolics (mg/GAE/100g) | 1.52  36.08 | 3.47  135.4 |

Av. CHO = Available carbohydrates. CB: control bread; BB: buckwheat bread

**Proximate composition of raw materials and bread samples (% dry basis)^*^**

| **Sample** | **Ash** | **Protein** | **Fat** | **Fiber** | **CHO** |
| --- | --- | --- | --- | --- | --- |
| Wheat flour | 1.75 ± 0.35^a^ | 11.59 ± 0.30^a^ | 1.25 ± 0.34^d^ | 2.00 ± 0.05^cd^ | 83.40 ±0.30^a^ |
| Buckwheat F | 1.02 ± 0.00^c^ | 8.75 ± 0.00^e^ | 2.75 ± 0.35^c^ | 5.25 ± 0.35^a^ | 82.25±0.70^b^ |
| WB | 1.25 ± 0.35^b^ | 10.50 ± 0.61^b^ | 5.23 ± 0.35^b^ | 1.50 ± 0.70^d^ | 81.50±0.08^b^ |
| 30% BWB | 1.02 ± 0.00^c^ | 9.02 ± 0.09^dc^ | 5.25 ± 0.35^b^ | 2.00 ± 0.00^cd^ | 82.54±0.26^b^ |
| 40% BWB | 1.01 ± 0.00^c^ | 9.71 ± 0.04^cd^ | 6.25 ± 0.35^a^ | 2.65 ± 0.21^bc^ | 80.38 ±0.60^c^ |
| 50% BWB | 1.03 ± 0.00^c^ | 10.16 ±0.04^bc^ | 7.05 ± 0.21^a^ | 3.35 ± 0.21^b^ | 78.42±0.46^d^ |

^*^Values are means ± SD of triplicate analyses.

Means in the same columns with different letters are significantly different (P < 0.05, LSD test).

WB: wheat bread, BWB: buckwheat-containing bread.

Amendola, J. and N. Rees, *Understanding baking: the art and science of baking*. 2003, John Wiley & Sons.
